# Structural analysis of human SEPHS2 protein, a selenocysteine machinery component, over-expressed in triple negative breast cancer

**DOI:** 10.1038/s41598-019-52718-0

**Published:** 2019-11-06

**Authors:** Carmine Nunziata, Andrea Polo, Angela Sorice, Francesca Capone, Marina Accardo, Eliana Guerriero, Federica Zito Marino, Michele Orditura, Alfredo Budillon, Susan Costantini

**Affiliations:** 1 Experimental Pharmacology Unit, Laboratori di Mercogliano, Istituto Nazionale Tumori – IRCCS – Fondazione G. Pascale, Napoli, Italia; 2 Department of Mental and Physical Health and Preventive Medicine, Università degli Studi della Campania “Luigi Vanvitelli”, Pathology Unit, Napoli, Italia; 3 Istituto Tecnico Industriale (ITIS) “Guido Dorso”, Avellino, Italia; 4 Division of Medical Oncology, Department of Precision Medicine, School of Medicine, Università degli Studi della Campania “Luigi Vanvitelli”, Napoli, Italia

**Keywords:** Bioinformatics, Cancer, Protein structure predictions

## Abstract

Selenophosphate synthetase 2 (SEPHS2) synthesizes selenide and ATP into selenophosphate, the selenium donor for selenocysteine (Sec), which is cotranslationally incorporated into selenoproteins. The action and regulatory mechanisms of SEPHS2 as well as its role in carcinogenesis (especially breast cancer) remain ambiguous and need further clarification. Therefore, lacking an experimentally determined structure for SEPHS2, we first analyzed the physicochemical properties of its sequence, modeled its three-dimensional structure and studied its conformational behavior to identify the key residues (named HUB nodes) responsible for protein stability and to clarify the molecular mechanisms by which it induced its function. Bioinformatics analysis evidenced higher amplification frequencies of SEPHS2 in breast cancer than in other cancer types. Therefore, because triple negative breast cancer (TNBC) is biologically the most aggressive breast cancer subtype and its treatment represents a challenge due to the absence of well-defined molecular targets, we evaluated SEPHS2 expression in two TNBC cell lines and patient samples. We demonstrated mRNA and protein overexpression to be correlated with aggressiveness and malignant tumor grade, suggesting that this protein could potentially be considered a prognostic marker and/or therapeutic target for TNBC.

## Introduction

The selenoproteins include selenocysteine (Sec), which is a nonstandard amino acid in the UGA codon. These proteins are found in all organisms throughout the tree of life. The incorporation of this 21st amino acid into proteins in mammalian cells is guided by the Sec biosynthesis machinery^1^, of which selenophosphate synthetase 2 (SEPHS2) is an important component^2^. SEPHS2 catalyzes the synthesis of selenophosphate from selenide, adenosine triphosphate (ATP), and water and produces adenosine monophosphate (AMP) and inorganic phosphate. Selenophosphate is the selenium donor for Sec synthesis, which, in contrast to other amino acids, takes place on its own tRNA, tRNA^Sec^ ^3,4^. The selenocysteine incorporation machinery requires certain protein-protein and protein-RNA interactions to function and is guided by stem-loop structures localized in the 3′ untranslated regions of selenoprotein-encoding genes. Recently, SEPHS2 has been demonstrated to interact with selenocysteine synthase (SepSecS) and SEPHS1, which plays a nonessential role in selenoprotein metabolism^5^.

Although some papers have reported that a decrease or increase in selenoprotein expression can induce a cancer phenotype^6^, the role of selenoproteins in carcinogenesis and their mechanisms of action and regulation remain ambiguous and need further clarification. In fact, few data are available regarding changes in SEPHS2 expression in cancer. Recently, researchers demonstrated that SEPHS2 expression was significantly decreased in gastric cancer and para-carcinoma tissues^7^. Moreover, Maciel-Dominguez *et al*. (2013) evaluated whether the effects of selenium on gene expression were exerted through miRNAs in a human colon adenocarcinoma cell line (Caco-2)^8^ and demonstrated that miR-185 played a role in the upregulation of SEPHS2 expression the maintenance of selenoprotein synthesis^8^. SEPHS2 levels were reported to be decreased in BRCA1-linked breast cancer versus BRCA1-negative cancer^9^, although its expression in specific breast cancer subtypes is unknown. In fact, breast cancer is a heterogeneous cancer with three different molecular subtypes based on overexpression of the estrogen and progesterone receptors, overexpression of the epidermal growth factor receptor 2 (HER2) receptor or the absence of these three receptors^10^. This latter subtype is defined as TNBC. TNBC constitutes 10%–20% of all breast cancers, affects younger patients, has lymph node involvement at diagnosis, and is the most aggressive type^11^. Treatment of TNBC patients still represents a challenge due to the absence of well-defined molecular targets^10,11^.

Therefore, since no three-dimensional structure of this protein has been experimentally determined, we focused our attention first on the utility of determining the SEPSH2 structure and studying the related structure-fluctuation relationships to examine the putative molecular mechanisms through which it induced its function. Additionally, because a bioinformatics analysis of databases found higher amplification frequencies in breast cancer, we evaluated SEPHS2 expression in two TNBC cell lines (MDA-MB231 and MDA-MB468) and TNBC patients to elucidate whether this protein could be correlated with the initiation and progression of this breast cancer subtype.

## Results and Discussion

### Sequence analysis of human SEPHS2

**Figure 1:**
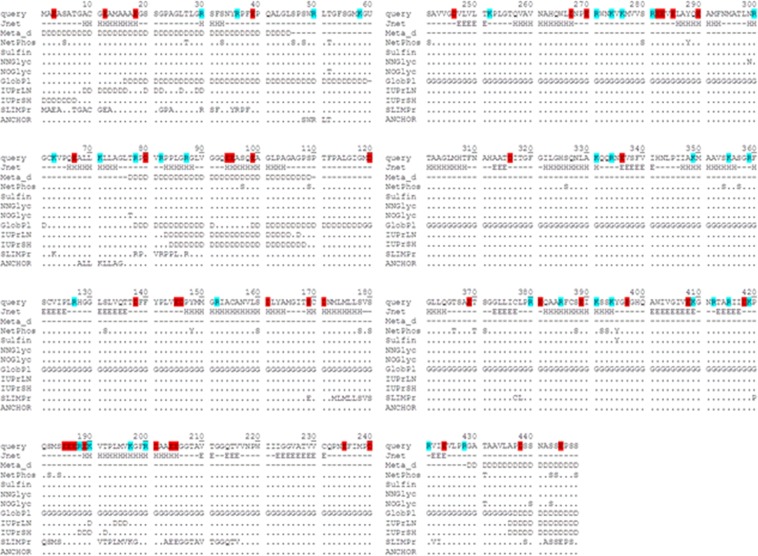
Sequence analysis of human SEPHS2. The negatively and positively charged residues are shown in red and cyan, respectively. We report predictions for secondary structure (Jnet), disorder propensity (Meta_d and IUPred), globularity propensity (GlobPL), predicted and experimentally determined phosphorylation sites (NetPhos and Phosphosite), sulfination sites (Sulfinator), glycosylation sites (NetNGlyc and NetOGlyc), and molecular recognition features (MorfPred and ANCHOR).

First, the human SEPHS2 sequence (UniProt code: Q99611) was analyzed to identify the relationships between its amino acid composition and conformation and particularly to assess how the amino acid composition of SEPHS2 could affect the conformational propensity to form regular secondary structures or be disordered using the ProtParam tool^12^. The sequence is characterized by 27 prolines, 48 glycines and 43 negatively (*f−* = 0.096) and 36 positively (*f*+ = 0.080) charged residues (Fig. 1). SEPHS2 is located in Region 1 of the state diagram, which contains weak polyampholytes and polyelectrolytes. The fraction of charged residues (FCR) in the protein is <0.25, the net charge per residue (NCPR) is <0.25, and the protein has a propensity to form globule and tadpole ensembles in agreement with Das and Pappu^13^ (Supplementary Fig. 1).

The secondary structure prediction by Jnet^14^ suggested the putative presence of 15 α-helices, 12 β-strands and some disordered regions. This finding was confirmed by the prediction of putative disordered regions by Genesilico MetaDisorder, GlobPlot and IUPred^15^. Some regions with high disorder propensities are located at the N-terminal (positions 1–60 and 77–110) and C-terminal regions (positions 428–448) (Fig. 1).

Since posttranslational modifications are important for the functional features of intrinsically disordered proteins (IDPs), including modulating the recognition of molecular partners^16^, we evaluated the putative presence of phosphorylation, sulfation and glycosylation sites as well as linear motifs associated with protein interactions. As shown in Fig. 1, analysis of the SEPHS2 sequence revealed 31 phosphorylation sites predicted by NetPhos^17^. Among these sites, four located at positions 33, 46, 97 and 109 have also been found experimentally, as shown by the PhosphoSitePlus analysis^18^. Sulfation prediction by the Sulfinator tool^19^ showed only one sulfation site at position 395, whereas the prediction of glycosylation sites by the NetNGlyc and NetOGlyc tools^20^ indicated a potential N-linked site at position 299 and five O-linked sites at positions 52, 77, 431, 440 and 443. The prediction of binding regions in disordered proteins by the ANCHOR^21^ and α-MoRF-PredII^22^ tools indicated potential binding positions in regions 1–80, 170–218, 379–380 and 420–448.

All these analyses demonstrated that three regions of SEPHS2 with high disordered propensities (positions 1–60, 77–110 and 428–448) contained posttranslational modifications that could induce a conformational change on the SEPHS2 structure with their charges.

### Molecular modeling of human SEPHS2

**Figure 2:**
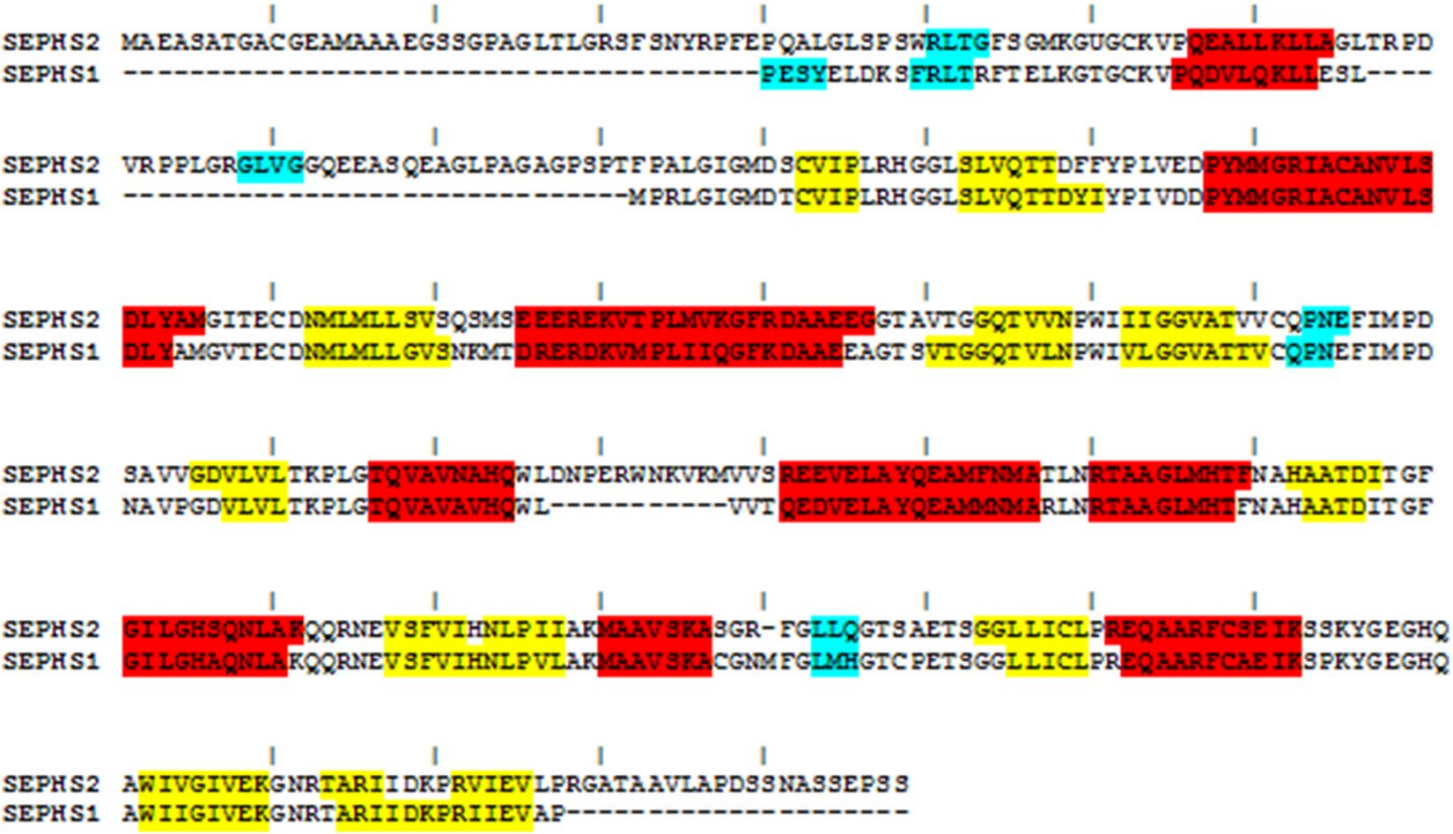
BLAST alignment between human SEPHS2 and human SEPHS1. The 3_10_-helices, α-helices and β-strands are shown in cyan, red and yellow, respectively.

The SEPSH2 structure was modeled by an integrated procedure based on comparative modeling and fold recognition that was described in our recent papers^23,24^. A BLAST search^25^ showed that region 41–427 of SEPHS2 had 75% sequence identity with human SEPHS1 (PDB code: 3FD5)^26^. No possible templates were available for the first 40 residues in the N-terminal region and the last 19 residues in the C-terminal region. Therefore, we created ten models for region 41–427 of SEPHS2 using the SEPHS1 structure as the template in the MODELLER program^27^ (Fig. 2) and analyzed their energetic and stereochemical quality. The best selected model showed 98.1% of residues in the allowed regions of the Ramachandran plot^28^ and an energetic Z-score^29^ of −9.01, demonstrating the good quality of the model. Then, this model was subjected to loop refinement using the MODELLER tool to energetically improve the conformation of the disordered 77–110 region loop. The final SEPHS2 model for the 41–427 region is composed of 3 3_10_-helices, 9 α-helices and 13 β-sheets. Comparison of the secondary structures between human SEPHS2 and SEPHS1 showed that the helices and β-strands were sufficiently conserved along the sequences, with only a few changes in their lengths (Fig. 2).

To gain further insights into the complete structure of SEPSH2, we modeled the N-terminal (1–40) and C-terminal (428–448) regions using the *“ab initio”* folding method with the MUSTER program^30^. The best 3D model of the N-terminal region had a Z-score of −0.26 and a total percentage of residues in the allowed regions of the Ramachandran plot of 97.4%, whereas that of the C-terminal region had a Z-score of −1.99 and 100% of residues in the favored region.

**Figure 3:**
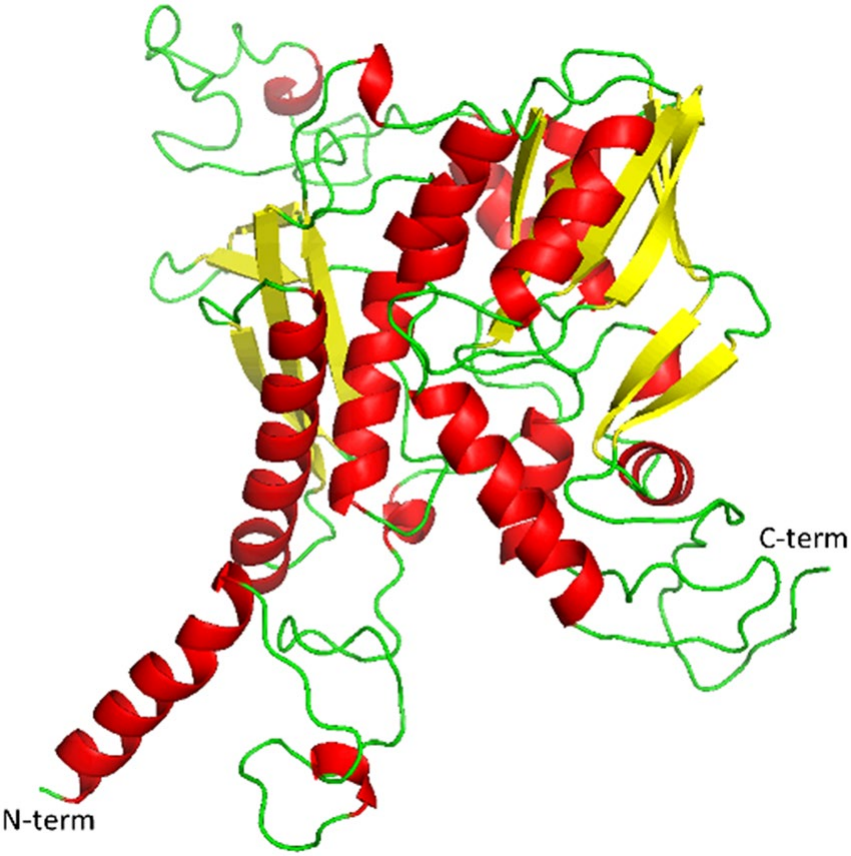
Complete SEPHS2 model obtained by the molecular modeling approach. In detail, 3_10_-helices and α-helices are reported in red, β-strands in yellow and loops in green.

Finally, we modeled the complete SEPHS2 structure using the three models reported above as templates for regions 1–40, 41–427 and 428–448. The 3D model of complete SEPHS2 had an energetic Z-score of −8.5 and 98.7% of the residues in the allowed regions. As shown in Fig. 3, the entire SEPHS2 model showed an N-terminal domain with an α-helix and a long disordered loop, a central core with an α−β 2-layer sandwich architecture and a disordered C-terminal domain.

Overall, these data highlighted that the SEPHS2 model conserved the structure of the SEPHS family. This finding was also confirmed by the circular dichroism spectrum analysis obtained from the protein atom coordinates by the PDB2CD tool (http://pdb2cd.cryst.bbk.ac.uk/). This analysis demonstrated overlap of the spectra and similarity of secondary structures related to our model and crystallographic structures of SEPHS1 from four different species (human, *Escherichia coli*, *Aquifexaeolicus* and *Leishmania major*) (Supplementary Fig. 2).

Subsequently, the whole model was subjected to molecular dynamics (MD) simulations at neutral and acidic pH in water by the GROMACS software^31^ to obtain more information on its structural dynamics features.

### Comparison between MD simulations at neutral and acidic pH

MD simulations were conducted at neutral and acidic pH to study the possible roles played by charged amino acids in SEPHS2 and the related electrostatic interactions. Importantly, an acidic extracellular pH is reported to be a major feature of cancer tissues^32^.

**Figure 4:**
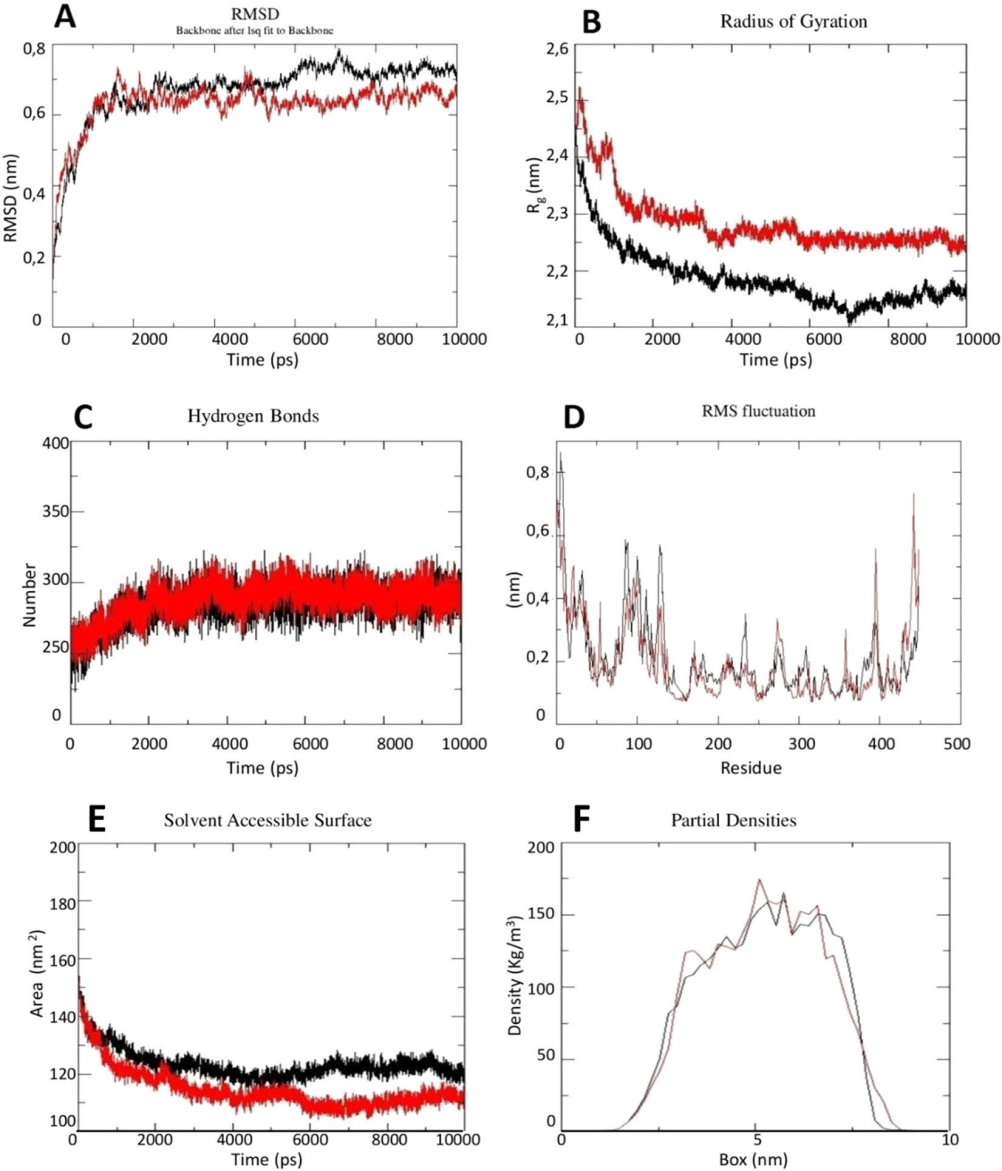
(**A**) RMSD, (**B**) gyration radius, (**C**) H-bond, (**D**) RMSF, and (**E**) solvent accessible surface plots and (**F**) partial densities for SEPHS2 during MD simulations at neutral (in red) and acidic (in black) pH.

The analysis of the trajectories demonstrated that the MD simulations at both neutral and acidic pH reached convergence after 2 ns **(**Fig. 4A**)**. However, compared to the MD simulation at neutral pH, the MD simulation at acidic pH reached (i) a greater compactness with a gyration radius (Rg) equal to 2.15 nm (Fig. 4B) and (ii) more stability due to an increase in the H-bond number (Fig. 4C) even if the protein floated more, as was visible in the root mean square fluctuation (RMSF) plot (Fig. 4D**)**. Moreover, evaluation of the solvent accessible area (ASA) (Fig. 4E), using protein a-sphericity as an indirect measure, showed that SEPHS2 tended to be more compact at acidic pH and tended to move toward the center of the box (Fig. 4F).

Analysis of the pattern of secondary structure evolution (Supplementary Fig. 3) suggested that most of the helices and β-strands remained relatively stable during MD simulation at neutral pH, with the exception of some terminal residues in short helices that tended to lose their regular structure. On the other hand, during the simulation at acidic pH, the secondary structure evolution of SEPHS2 revealed an increase in structural fluctuations compared to those found at neutral pH; in fact, some short α-helices and β-strands were completely lost during the simulation (Supplementary Fig. 3).

**Figure 5:**
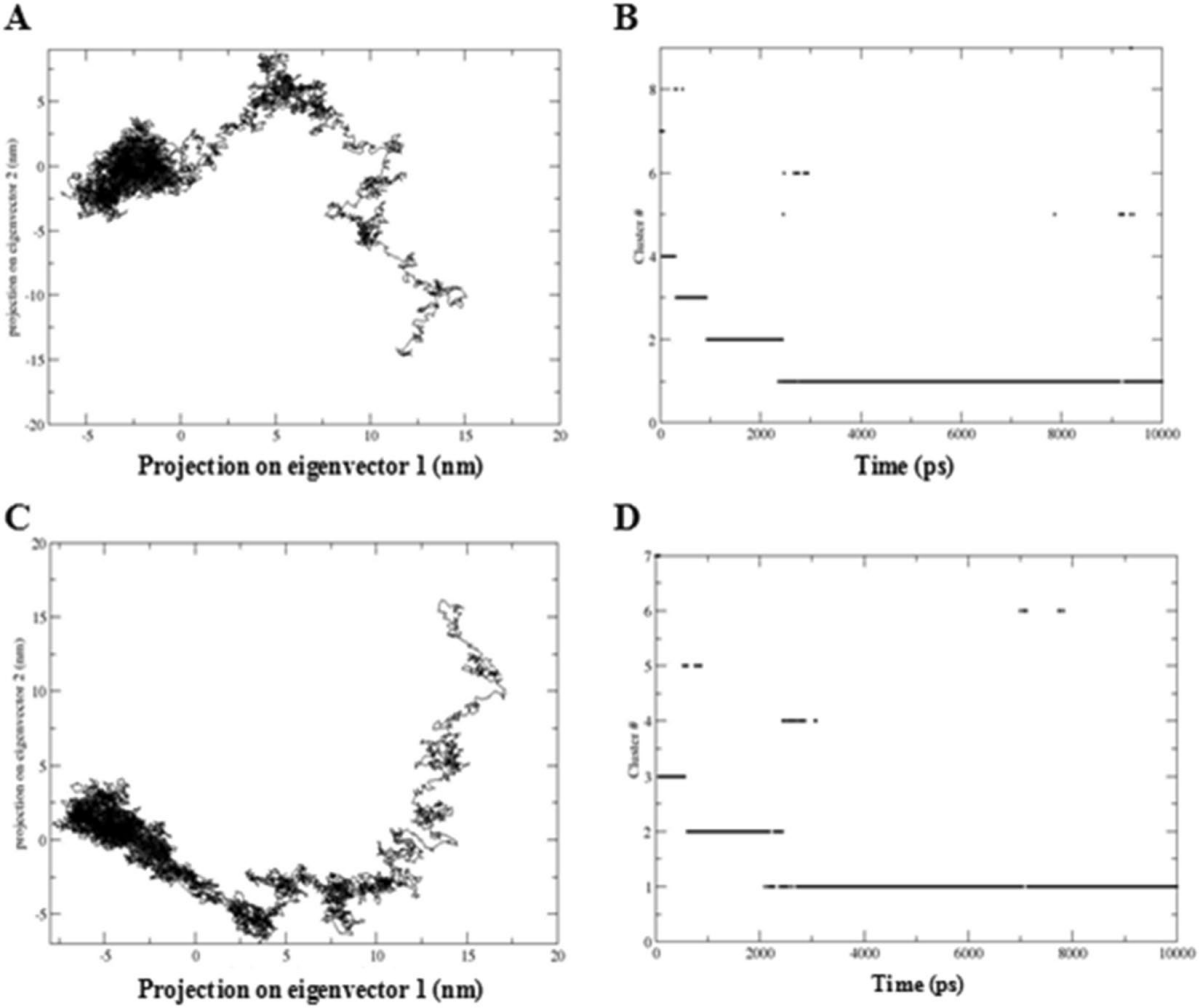
Projection plot and cluster analysis results for SEPHS2 during MD simulations at neutral (**A,B**) and acidic pH (**C,D**), respectively.

Subsequently, to better understand the collective fluctuations of SEPHS2 during the MD simulations, we performed principal component analysis (PCA), cluster analysis, and covariance matrix analysis. Specifically, the projections of the principal eigenvectors were plotted onto the plane over the Cα coordinates, and cluster analysis was performed based on the root mean square deviation (RMSD) values between the different conformations by selecting structures sharing similar conformational features during the simulation. The PCA plot and covariance matrix showed that during the MD simulation at acidic pH: (i) the atoms of SEPHS2 moved much faster and favored conformational interchanges to reach increased structural stability (Fig. 5); and (ii) the fluctuating regions, indicated by a more intensive red color, were located at both the N-terminal and C-terminal regions, which included negatively charged residues (Supplementary Fig. 4). The cluster analysis showed the presence of eight and seven clusters, among which three and two were the most populated at neutral and acidic pH, respectively (Fig. 5). However, although the total number of clusters was similar in the simulations at neutral and acidic pH, the transition rate of the conformational interchange among clusters was very high at the acidic pH, indicating that more fluctuations were necessary to reach a compact structure.

Finally, we focused on residue Sec. 60. During the MD simulation at acidic pH, this residue was more exposed on the surface and interacted with Lys 58, Cys 62 and Arg 359 during all simulations.

### Identification of key residues (HUB) by residue interaction network (RIN) analysis during MD simulations at neutral and acidic pH

For studies of the specific role of individual residues in the general organization of a protein, RIN analysis is a very useful method to identify residues with the strongest coordinating role (i.e., HUB residues) and to obtain information about protein stability^23,24^. Our model was dynamically stabilized by a certain number of H-bonds and interactions with the closest atoms (IACs) as well as by salt bridges and π-cation and π-stacking interactions during the MD simulations at neutral and acidic pH based on evaluation with the Protein Interactions Calculator (PIC)^33^, HBPLUS^34^ and COCOMAPS^35^ tools (Table 1). In detail, the RIN analysis was performed on the SEPHS2 conformation at 0, 2, 4, 6, 8 and 10 ns of simulation time by evaluating the following seven topological parameters to identify HUB nodes: (i) density of the maximum neighborhood component (DMNC), (ii) degree, (iii) maximal clique centrality (MCC), (iv) maximum neighborhood component (MNC), (v) betweenness, (vi) bottleneck, and (vii) closeness^36,37^.

**Table 1:** Number of H-bonds (subdivided into main chain - main chain (MM), main chain - side chain (SM), side chain - side chain (SS) and side chain - main chain (SM)), π-cation and π-stacking interactions, salt bridges and interactions of closest atoms (IACs) during MD simulations in water at neutral and acidic pH.

Neutral pH	H-bonds				π-cation	π-stacking	Salt bridges	IAC
	MM	MS	SS	SM				
0 ns	199	20	24	35	7	4	7	1990
2 ns	218	33	34	30	3	5	7	2202
4 ns	230	28	28	36	2	6	6	2235
6 ns	217	42	21	39	1	4	5	2238
8 ns	244	31	27	29	1	3	6	2264
10 ns	227	38	25	41	2	4	5	2241
**Acidic pH**								
0 ns	205	23	19	32	7	5	11	1990
2 ns	222	21	14	39	8	2	5	2330
4 ns	222	29	16	36	11	4	5	2354
6 ns	233	27	12	37	8	4	3	2360
8 ns	229	27	15	37	8	3	6	2389
10 ns	228	25	11	38	7	4	6	2351

**Figure 6:**
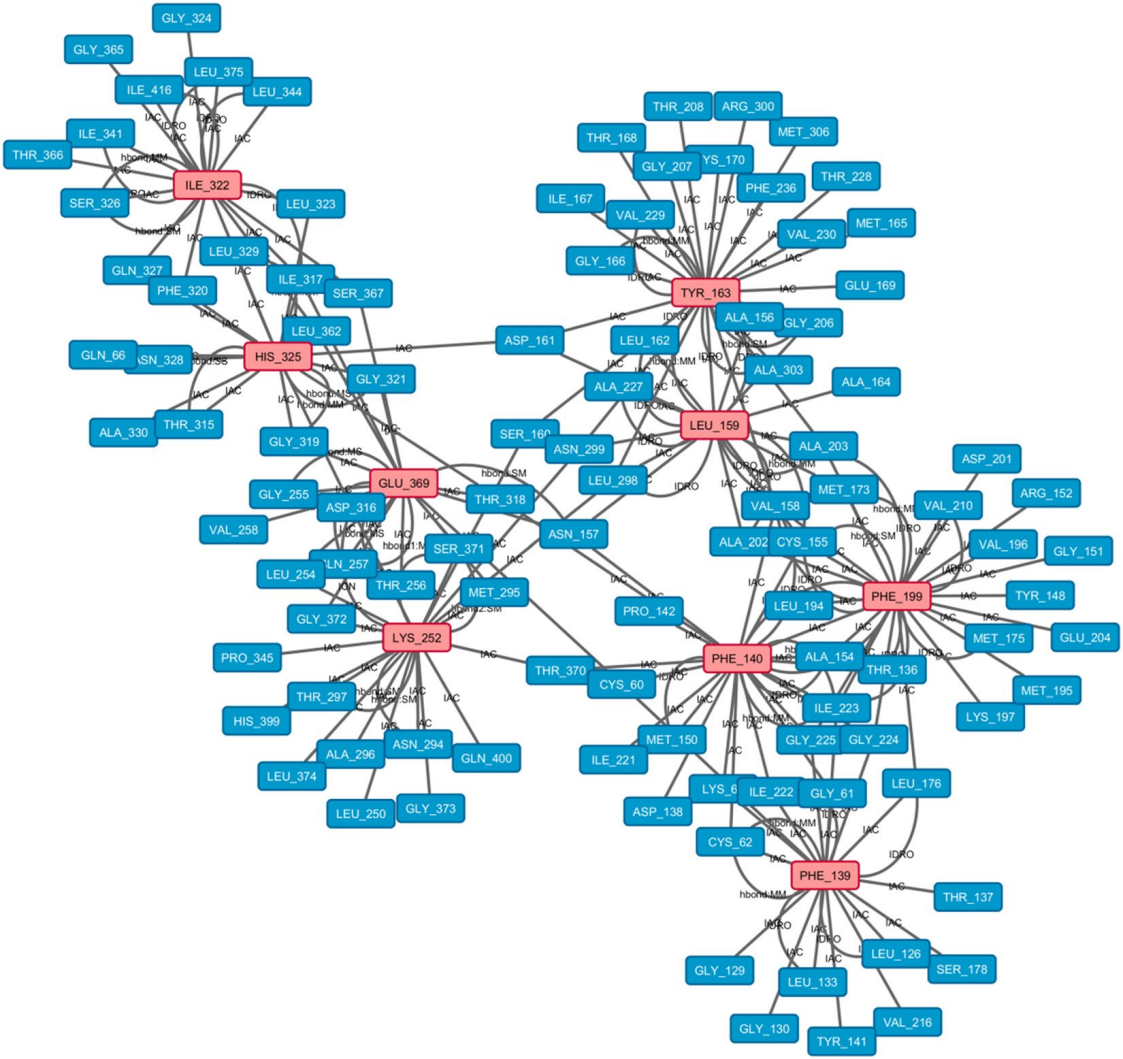
Residue interaction network for SEPHS2 at the end of the neutral pH simulation. HUB nodes are in pink, and the other residues are in light blue.

This analysis showed that in the MD simulation at neutral pH (Fig. 6**)**, Phe 199 was the most conserved HUB residue during the simulation based on five out of seven centrality measures, suggesting that this residue might play an important structural role during MD evolution. Moreover, the analysis identified the following HUB residues: Lys 252 after 2, 4 and 8 ns of simulation, Tyr 163 after 4, 6 and 10 ns, and Phe 140 after 6, 8 and 10 ns. These data suggested that Lys 252, Tyr 163 and Phe 140 followed the conformation changes of the protein during the simulation at neutral pH and played a role in stabilizing the protein structure (Table 2**)**. In fact, during the MD simulation, these four HUB residues tended to form a higher number of H-bonds (Supplementary Table 1) and to become more buried, as shown by the ASA analysis (Supplementary Table 2), confirming their roles in promoting the compactness of this protein.

**Table 2:** Differences in residue interaction network (RIN) analysis of SEPHS2 during MD simulations in water at neutral and acidic pH.

Neutral pH	0 ns	Residue interaction network				10 ns
		2 ns	4 ns	6 ns	8 ns	
	Ile 322	Lys 252	His 325	Phe 140	Phe 140	Phe 139
	Phe 199	Phe 199	Leu 159	Tyr 163	Glu 369	Phe 140
			Lys 252	Phe 199	Ile 322	Phe 199
			Phe 199		Lys 252	Tyr 163
			Tyr 163		Phe 199	
**Acidic pH**		Phe 320	Phe 199	Phe 199	Phe 199	Phe 199
		Glu 283	His 325	Phe 320	Phe 140	Val 158
		Lys 190	Met 295	Phe 140	Phe 320	Asn 157
			Phe 140			
			Phe 360			

**Figure 7:**
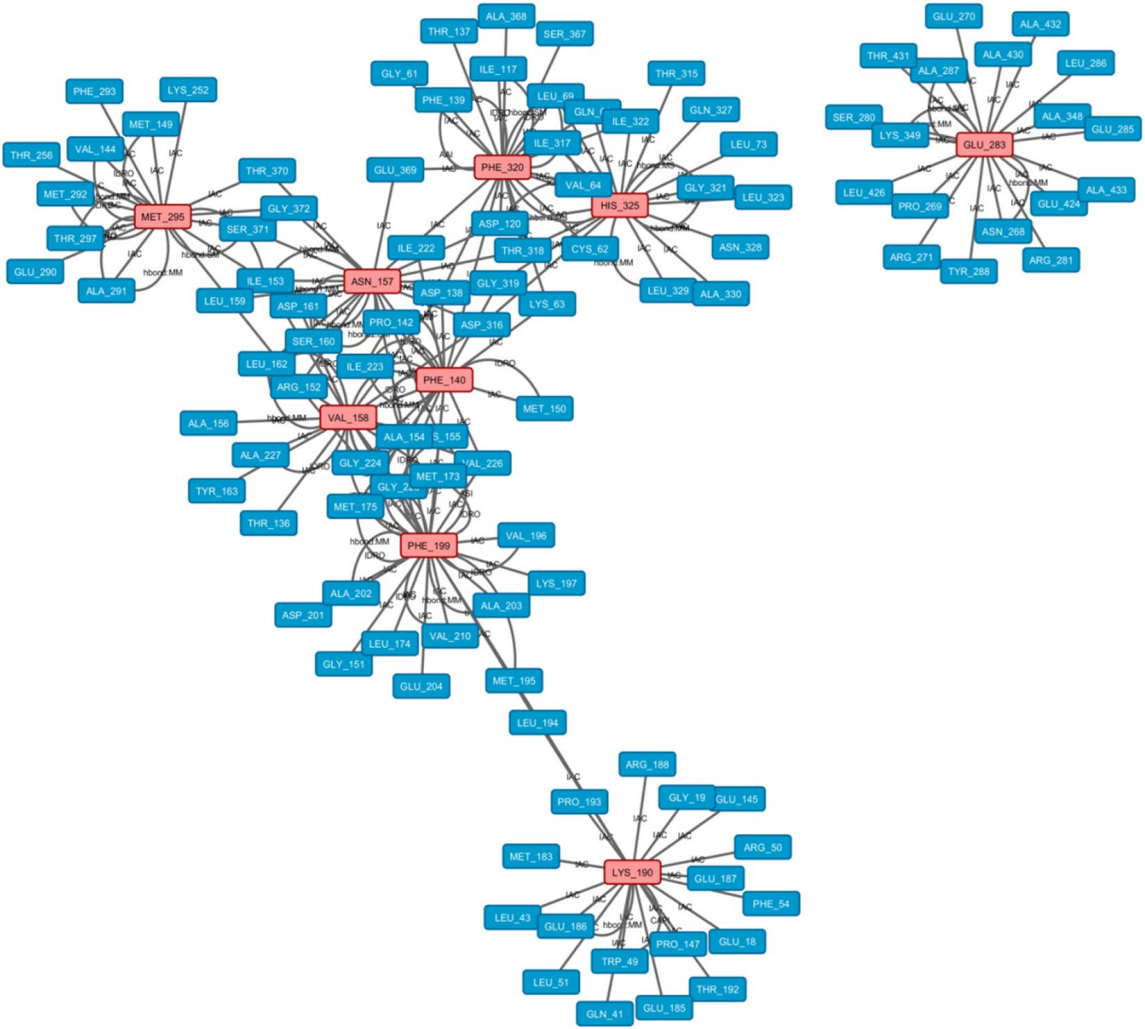
Residue interaction network for SEPHS2 at the end of the acidic pH simulation. HUB nodes are in pink, and the other residues are in light blue.

By analyzing the network obtained for MD simulation at acidic pH (Fig. 7**)**, we showed that no HUB node was identified at 0 ns, whereas nine residues (Phe 320, Glu 283, Lys 190, Phe 199, His 325, Met 295, Phe 140, Val 158 and Asn 157) were considered HUB nodes during the rest of the simulation (Table 2). Among these residues, an increase was observed in the formation of H-bonds during the simulation for Phe 320, Glu 283 and Met 295 (Supplementary Table 1), whereas a decrease in ASA values was observed for Phe 320, Glu 283, Lys 190, Phe 199, His 325 and Phe 140. These findings confirmed that these latter residues were buried during the simulation and tended to play a structural role (Supplementary Table 2).

Then, to examine whether these HUB nodes created subnetworks able to stabilize the structure, we focused on the interactions between them. As shown in Figs 6 and 7, the pivotal HUB residues formed IAC, π-cation and hydrophobic interactions and H-bonds during the MD simulations at neutral and acidic pH, confirming a structural role for these HUB nodes in driving the conformational organization of SEPHS2 for all MD simulations and in maintaining its compactness.

Moreover, to understand the possible biological relevance of these HUB residues, we evaluated whether they were part of the active site composed by Sec. 60, Lys 63 and 319–325 region. In fact, it is notable that (i) Phe 320 (a HUB residue at acidic pH), an Ile 322 (HUB residue at neutral pH) and a His 325 (HUB residue at neutral and acidic pH) were present in the active site; (ii) His 325 formed a H-bond with Gly 319 of the active site; and (iii) Ile 322 formed a hydrophobic interaction with Leu 323. However, the other HUB residues interacted with the active site by proximity interactions (IAC), suggesting a potential molecular advantage of the HUB residues in stabilizing the active site and permitting SEPHS2 to develop its activity.

To verify if there were mutations of HUB residues reported in the SNP databases and correlated with breast cancer and/or the TNBC subtype, we interrogated different online databases containing SNPs such as dbSNP (https://www.ncbi.nlm.nih.gov/snp/), CbioPortal (http://cbioportal.org)^38,39^ and COSMIC (https://cancer.sanger.ac.uk/cosmic). We found 1252 SNPs associated with SEPHS2 by dbSNP database, and in particular, we found one missense mutation of Tyr163 (rs1284481836) and Phe140 (rs770633929), two missense mutations (rs1366381318, rs746372646) of Met295 and four missense mutations (rs376948016, rs1446115907) of Asn157. However, all of them are not still associated with a pathologic state and no clinical significance was still evidenced. Moreover, we searched the CbioPortal and cosmic databases for mutations of the residues correlated with breast cancer, particularly, the TNBC subtype. In detail, we found three missense mutations (Val404Ile, Met183Ile and Phe360 Val) correlated with the breast invasive carcinoma by CbioPortal, and four missense mutations (and Gly358Glu) were correlated to the breast ductal carcinoma by COSMIC. No specific mutations in HUB residues were found to correlate to the TNBC subtype.

In conclusion, all these data related to RINs at neutral and acidic pH revealed the following: (i) three HUB residues (two at neutral pH and two at acidic pH) were present in the active site; (ii) at acidic pH, a greater number of the HUB residues were buried, increasing the compactness of the structure; and (iii) these buried residues included three residues, Met 295, Phe 140 and Asn 157, reported to be mutated by the dbSNP database, which suggests that at acidic pH, their mutation can decrease the compactness of the structure and induce conformational fluctuations and/or changes to the biological activity of SEPHS2.

Therefore, it could be useful to focus research on understanding the role of this protein in cancer development and progression.

### SEPHS2 involvement in cancer based on TCGA datasets

To search for information related to SEPHS2 involvement, we first analyzed data in TCGA databases related to SEPHS2 amplification and mutation frequencies in cancer with the CbioPortal tool (http://cbioportal.org)^38,39^. As shown in Supplementary Table 3, higher amplification frequencies were found in breast cancer.

Considering that TNBC represents the most aggressive breast cancer subtype and that its specific molecular targets have not been well defined, we evaluated SEPHS2 expression in TNBC cell lines and patient tissues.

### SEPHS2 expression in TNBC cells and tissues by RT-qPCR

**Figure 8:**
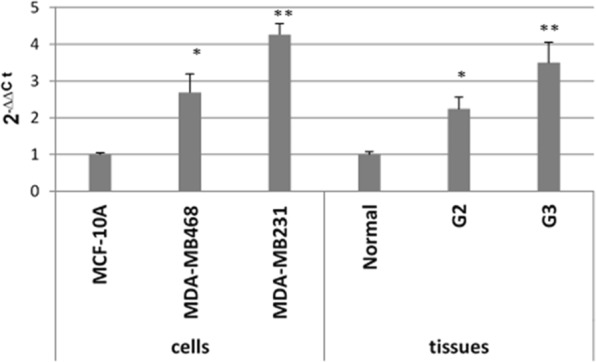
SEPHS2 levels in MDA-MB468 and MDA-MB231 cells compared to MCF10A cells and in TNBC tissues compared to their normal counterparts based on RT-qPCR analysis. The p-values <0.05 and <0.01 are indicated by * and **, respectively.

SEPHS2 expression was evaluated using RT-qPCR in two TNBC cell lines (MDA-MB231 and MDA-MB468), normal breast cells (MCF-10A), and thirty TNBC tissues and their adjacent normal breast tissue counterparts (Table 3). As shown in Fig. 8, the SEPHS2 level (expressed as 2^-ΔΔCt^) was higher in the cell lines MDA-MB231 (p-value < 0.01) and MDA-MB468 (p-value < 0.05) cell lines than in the MCF-10A cells and in the MDA-MB231 cells than in the MDA-MB468 cells (Fig. 8). The difference between these two cell lines is certainly due to their different phenotypic characteristics^40^. In fact, both MDA-MB231 and MDA-MB468 cells are poorly differentiated TNBC cells with mutations in p53. MDA-MB231 cells also present mutations in BRAF and KRAS, low levels of claudin-3, claudin-4, claudin-7, E-cadherin and proliferation marker Ki67, and high levels of vimentin and CD44^+^CD24^-^, which are markers associated with the epithelial-mesenchymal transition and mammary cancer stem cells (CSCs), respectively. Moreover, MDA-MB468 cells have mutations in PTEN and high levels of EGFR^+^, cytokeratin 5/6^+^ and Ki67 gene expression^40^. In this context, MDAMB231 cells are more invasive^40^, which may explain why the SEPHS2 levels are higher in MDA-MB231 cells than in MDA-MB468 cells.

**Table 3:** Clinicopathological assessment of the patients.

	Number of patients
***Female***	30
***Age (mean ± st.dev.)***	52.6 ± 13.5
***ER-***	30
***PR-***	30
***HER2-***	30
***Primitive TNBC***	30
***Histotype***	
No special type (ductal)	28
Lobular carcinoma	1
Metaplastic carcinoma	1
***Grading***	
G2	10
G3	20
***Ki67***	
<50%	15
≥50	15
***Status***	
Live	25
Dead	5

Moreover, in accordance with the cellular results, the SEPHS2 level was increased in the TNBC tissues of with grade 2 (p-value < 0.05) and grade 3 (p-value < 0.01) compared to that in their normal counterparts and, in particular, increased with the malignant grade (Fig. 8).

To confirm these data at the protein level, we subjected the collected tissues to SEPHS2 staining by immunohistochemistry (IHC).

### IHC of TNBC tissues

Microscopically, a solid architecture of the TNBC tissues was observed, with little or no formation of tubular structures and a poor intercellular stroma. Some carcinomas showed medullary-like features with lymphocyte infiltration on the periphery of the neoplastic population. In other cases, cellular cords associated with necrotic areas were observed. The neoplastic cells showed a high nucleus/cytoplasm ratio, evident nucleoli and a high mitotic index. Infiltrative lobular carcinoma showed a classical and diffuse mixed growth pattern with pleomorphic cells. Metaplastic carcinoma showed giant cells and areas with squamous metaplasia.

**Figure 9:**
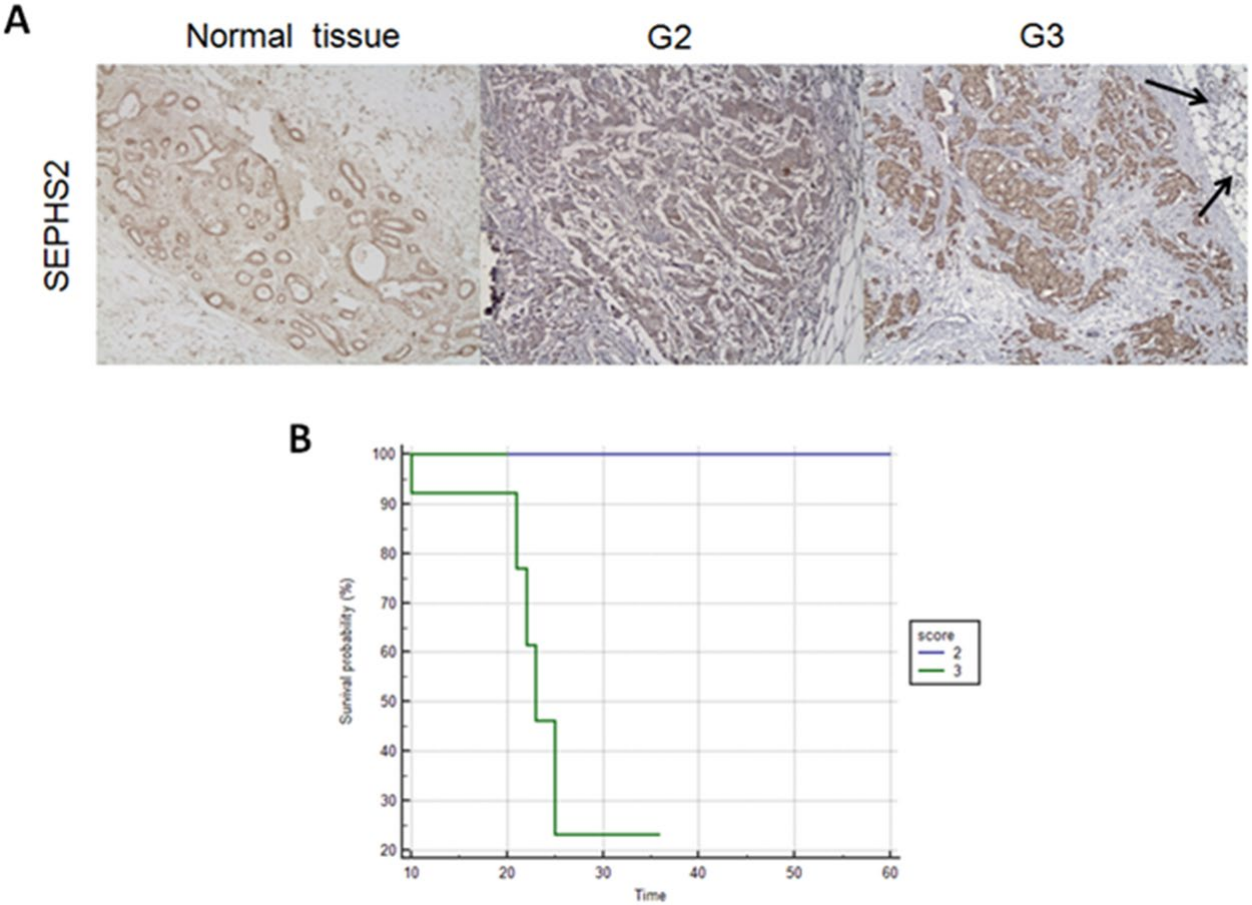
(**A**) Immunohistochemical observation of human SEPHS2 expression at 200x magnification in normal mammary tissues and grade 2 (G2) and 3 (G3) TNBC patients. In the panel related to a tissue section from a G2 patient, only the neoplastic lesion is visible. In the panel related to a tissue section from a G3 patient, two black arrows indicate a lobular ductule of a normal mammary gland immersed in the adipose stroma; moreover, clusters of neoplastic cells are found in the same stroma. (**B**) Kaplan–Meier curves of the overall survival of TNBC patients. Blue line: score = 2 and green line: score = 3.

All the TNBC samples were immunoreactive for SEPHS2, with 26 cases higher than 30% and the other 4 cases between 5 and 30%. The positivity was expressed both at the cytoplasmic and nuclear levels, with a staining intensity ranging from weak to moderate. In context, a more diffuse and moderate intensity (score = 3) was observed in poorly differentiated TNBC patients with grade 3 than in patients with grade 2, for whom weak staining (score = 2) was evident (Fig. 9A). In detail, (i) for 80% of patients (8 of 10) with grade 2, a score = 2 has been assigned (chi-square test, df = 6.316 and p-value = 0.0120); (ii) for 85% of patients (17 of 20) with grade 3, a score = 3 has been assigned (chi-square test, df = 4.787 and p-value = 0.0287). The “*in situ*” carcinomatous component was less than 25% and showed no significant differences in immunoreactivity from the infiltrating components. As shown in Fig. 9A, the healthy glandular component also expressed extremely weak cytoplasmic positivity (score = 1) at the level of myoepithelial cells.

Overall, these evaluations suggest that the gradual increase in SEPHS2 expression is associated with an increased malignant grade in TNBC patients. In fact, higher SEPHS2 levels correlate with poor overall survival (with a p-value = 0.0067) (Fig. 9B).

## Conclusions

In this paper, we obtained for the first time a structural model of the SEPHS2 protein, which is an important component of the Sec biosynthesis machinery for which no three-dimensional structure has been experimentally determined. The obtained model consists of an N-terminal domain with an α-helix and a long disordered loop, a central core with an α−β 2-layer sandwich architecture and a disordered C-terminal domain. MD simulation of the SEPHS2 structure at neutral pH highlighted that the structure was stabilized through formation of π-cation interactions and H-bonds and showed that Sec. 60 remained exposed on the surface throughout the MD simulation, whereas other residues, termed HUB residues, tended to stabilize the structure. During the simulation at acidic pH, the stability and compactness of SEPHS2 were greater than those during the simulation at neutral pH, and Sec. 60 was more exposed on the surface. Hence, considering that acidic extracellular pH is a major feature of cancer tissues^32^, these structural features of SEPHS2 at acidic pH can help explain how this protein functions in cancer. Therefore, we suggest the utility of further experimental studies on these identified HUB residues to understand their specific roles in dysregulation of the Sec biosynthesis machinery in cancer.

We also highlighted for the first time that the SEPHS2 levels were higher in TNBC than in normal epithelial breast tissues and that the levels increased with the malignant grade, suggesting that this protein could be a potential novel prognostic marker and/or therapeutic target. Further studies are warranted to validate SEPHS2 as a prognostic marker in larger cohorts of TNBC patient samples.

Notably, selenoproteins can be secreted and detected in the blood^41^. Blood plasma and its proteins are an ideal source of biomarkers, since they represent a snapshot of a patient’s pathophysiological state at a given time. Indeed, so-called “liquid biopsies” allow dynamic monitoring with an insight into the process of spatial and temporal clonal evolution of the tumor, including secondary resistance to treatment, which is denied by the invasiveness of tissue biopsies. In the literature, it is reported that thioredoxin reductase 1 (TrxR1), a cytoplasmic and nuclear selenoprotein, is secreted and that its serum levels are associated with poor prognosis in non-small cell lung cancer^42^. Hence, to verify whether SEPHS2, a cytoplasmic protein, is also secreted, our laboratory will perform further studies on biological fluids.

## Methods

### Sequence analysis

The ProtParam tool was used to analyze the amino acid composition of the human SEPHS2 sequence (UniProt code: Q99611)^12^, and Jpred^14^ and MetaDisorder server^15^ (http://iimcb.genesilico.pl/metadisorder/) were used to predict the propensity to form regular secondary structure elements or to be disordered.

The charge distribution on the human SEPHS2 sequence was evaluated in agreement with Das and Pappu^13^. In particular, the FCR and NCPR were calculated using FCR = |*f*_+_ + *f*_*−*_| and NCPR = | *f*_+_ − *f*_*−*_|, where *f*_+_ and *f*_*−*_ represent the fractions of positive and negative charges, respectively. This calculation allows classification of the protein sequences in the following four regions of the state diagram: (i) Region 1 (FCR < 0.25 and NCPR < 0.25), which contains weak polyelectrolytes and polyampholytes and shows a tendency to form tadpole and globule ensembles; (ii) Region 2 (0.25 ≤ FCR ≤ 0.35 and NCPR ≤ 0.35); (iii) Region 3 (FCR > 0.35 and NCPR ≤ 0.35) which contains strong polyampholytes and has a tendency to form ensembles of hairpins, coils and chimeras; and (iv) Region 4 (FCR > 0.35 and NCPR > 0.35), which contains strong polyelectrolytes and tends to form ensembles of swollen coils^13^.

Posttranslational modifications, such as sulfation, phosphorylation and glycosylation, were predicted by the Sulfinator^19^, NetPhos^17^, and NetNGlyc and NetOGlyc^20^ tools, respectively. We also searched for experimental phosphorylation sites using the PhosphoSitePlus server^18^. Finally, the binding regions in disordered proteins were predicted by the ANCHOR^21^ and α-MoRF-PredII^22^ tools. All these procedures were performed in accordance with the relevant guidelines and regulations.

### Molecular modeling

The SEPSH2 structure was modeled using an integrated procedure based on comparative modeling and fold recognition that we described previously^23,24^. BLAST analysis^25^ showed that the 41–427 region of SEPSH2 had 73% sequence identity with human SEPHS1. Hence, human SEPHS1 was used as a starting template. We created ten structures using the MODELER program^27^ and selected the best model based on the energetic and stereochemical quality. In detail, the structures were analyzed with the ProSA^29^ and Ramachandran Plot 2.0^28^ tools to calculate the energetic stability (Z-score) and the numbers of residues in allowed and disallowed positions in the Ramachandran plot, respectively. The best selected model was subjected to a loop refinement tool to obtain a better structure of the unstructured disordered loop regions.

The N-terminal (1–40) and C-terminal (428–448) regions were modeled by MUSTER, which is a fold recognition tool based on a sequence profile-profile alignment algorithm (PPA)^30^.

Then, the complete 3D structure of SEPHS2 was modeled using as reference the models obtained, as reported above, for the N-terminal, C-terminal and 41–427 regions. The complete best model was chosen always by evaluating the energetic quality using the ProSA program^29^ and the stereochemical quality using the Ramachandran plot^28^.

The final SEPHS2 model was deposited in the ModelArchive database (10.5452/ma-y6ovo).

### MD simulations and analysis

The complete SEPHS2 model was subjected to MD simulations by the GROMACS software (v4.5.6)^31^, at neutral and acidic pH, for 10 ns at 300 K, using GROMOS43a1 as the force field. A cubic box (88.6 × 88.6 × 88.6 Å^3^) was designed to contain the model and covered with 6775 SPC216 water molecules.

Initially, 2000 energy minimization and 25000 position restraint steps were performed to minimize the protein system and the location of the water molecules.

The final trajectories were analyzed though different routines present in GROMACS, such as RMSD, gyration radius (R_g_), secondary structure evolution, RMSF, total solvent accessible area, PCA and partial densities.

For each conformer, the numbers of π-cations, H-bonds, π-stacking and IACs were calculated before MD and after 2, 4, 6, 8 and 10 ns of MD simulation using the PIC^33^, HBPLUS^34^ and COCOMAPS^35^ tools.

### Network analysis

Taking advantage of the NetworkAnalyzer and RINalyzer Cytoscape plugins, a network of interacting amino acids was created using as nodes the amino acids and as edges the π-cations, H-bonds, π-stacking and IACs to represent the interactions between them. The network topologies were analyzed by the CytoHubba plugin, and the HUB residues (the most correlated nodes) were identified. In detail, the following statistical network parameters were evaluated: (i) betweenness centrality; (ii) degree of nodes; (iii) bottleneck; (iv) closeness centrality; (v) DMNC; (vi) MNC and (vii) MCC^36,37^.

### Cancer evaluation using TCGA datasets

To determine the mutation and amplification frequencies of SEPHS2 in different cancers, we analyzed TCGA datasets using the CbioPortal tool (http://cbioportal.org)^38,39^.

### Cell culture

Normal human breast epithelial cells (MCF-10A) and two TNBC cell lines (MDA-MB231 and MDA-MB468) were used in this work. MDA-MB231 and MDA-MB468 cells were grown in RPMI 1640 (Lonza) supplemented with penicillin/streptomycin (100×) (Euroclone), fetal bovine serum (10%) (Invitrogen), and nonessential amino acids (100×) (Invitrogen), and Glutamax (100×) (Invitrogen) whereas MCF-10A cells in DMEM were supplemented with 20 ng/mL human epidermal growth factor (Life Technologies), 10 μg/mL human insulin (Life Technologies Corporation), and 0.5 μg/mL of hydrocortisone (Sigma-Aldrich). In the case of MDA-MB468 cells, Ham’s F-12 medium was also added to the medium (1:1 ratio). Finally, the cells were kept at 37 °C in an incubator in a humidified atmosphere (5% CO_2_ and 95% air).

### Tissue samples

We performed RT-qPCR and IHC on formalin-fixed paraffin-embedded (FFPE) blocks of TNBC tissues obtained by surgical resection from thirty patients. In this study, the adjacent normal breast tissue counterparts were used as tissue controls. This study was approved by the Ethics Committee of University of Campania. All the patients provided informed consent for study partecipation. The clinicopathological assessment of the patients is listed in Table 3. All thirty patients had primitive TNBC and did not present the progesterone and estrogen receptors, and HER2/neu genes. The tissues were obtained from 28 patients with ductal carcinoma, 1 with lobular carcinoma and 1 with metaplastic carcinoma. Moreover, 10 TNBC patients were grade 2 and 20 patients were grade 3. Five of these patients died.

### RNA preparation and RT-qPCR analysis of the cells and tissues

RNAeasy Mini Kit (Qiagen Inc) was used to extract total RNA from three cell lines. For the tissue samples, the paraffin was removed by xylene extraction for RNA isolation, and the Recoverall (TM) Total RNA Isolation Kit (Life Technologies-Ambion) was used to extract total RNA from tissue sections equivalent to 60 µm (three 20 µm sections). All the obtained RNA extracts were dissolved in diethyl pyrocarbonate-treated water, and their concentrations and purities were assessed by NanoDrop 2000 spectrophotometer (Thermo Scientific) at 260/280 nm of optical density. SuperScript VILO cDNA Synthesis Kit (Life Technologies Corporation) and nuclease-free water (Life Technologies-Ambion) were used to reverse-transcribe 2 µg of total RNA obtained for each sample and to dilute them, respectively.

Primer pairs for RT-qPCR were designed using the mRNA sequences deposited in the nucleotide data bank (NCBI) with an aim of obtaining a <100 bp amplicon. Oligonucleotides were obtained from Eurofins. The primer sequences were CATCCCCCTGAGGCACGG and TCACTCAGCACGTTGGCACA for SEPHS2 and TCTGGCACCACACCTTCTACAATG and AGCACAGCCTGGATAGCAAG for β-actin.

A Step-One Real Time PCR System (Applied Biosystems) was used to perform RT-qPCR experiments. An aliquot of cDNA (2 µL) was amplified in a mixture (25 µL) consisting of the reverse and forward primers (300 nM) and 2X SYBR Green PCR Master Mix (Applied Biosystems) using the following conditions: denaturation for 5 min at 95 °C, and, then, 44 cycles of denaturation for 30 s at 95 °C plus annealing for 1 min at 60 °C. Each reaction was performed in triplicate. β-Actin mRNA was used to normalize the data^43^ and Prism software (GraphPad Software) to perform statistical analysis (a paired t test). All these procedures were performed in accordance with the relevant guidelines and regulations.

### Immunohistochemistry of TNBC tissues

The sections were inserted in jars containing trisodium citrate solution (0.01 M) after alcohol rehydration and xylene dewaxing, microwaved, and, then, rinsed for 5 min in cool H_2_O and for 30 min at room temperature in H_2_O_2_ (3%). Successively, the samples were washed in Tris-buffered saline and incubated overnight at 4 °C with an anti-SEPHS2 antibody produced in rabbits (Sigma-Aldrich) using a 1:100 dilution. After incubation, biotinylated secondary antibodies plus streptavidin (Dako) were used to stain the samples, DAB chromogen (Dako) as substrate, and hematoxylin solution for nuclear counterstaining. All these procedures were performed in accordance with the relevant guidelines and regulations. However, we used some FFPE tissue samples of human ovarian cancer as positive control to verify the reliability of anti-SEPHS2 antibody, as suggested by the Sigma-Aldrich data sheet.

Immunoreactivity was evaluated in terms of positive stained cancer cellular percentage and staining intensity as described by Sinicrope *et al*.^44^. The staining intensity was scored in the following way: 1, extremely weak; 2, weak; and 3, moderate. The statistical significance was calculated by the Fisher and chi-square tests. Survival curves were evaluated and compared by log-rank test and the Kaplan–Meier method.

## Supplementary information

Supplementary Material
