# Supplementary material for: Structural analysis of human SEPHS2 protein, a selenocysteine machinery component, over-expressed in triple negative breast cancer
Source: Sci Rep. 2019 Nov 6;9:16131. doi: 10.1038/s41598-019-52718-0 (PMC6834634; doi:10.1038/s41598-019-52718-0)
Supplement: Supplementary file 1 — Supplementary Material [file 41598_2019_52718_MOESM1_ESM.docx]

**Structural analysis of human SEPHS2 protein, a selenocysteine machinery component, over-expressed in triple negative breast cancer**

**Carmine Nunziata***^1,^***^#^, Andrea Polo***^1,^***^#^, Angela Sorice***^1,^***^#^, Francesca Capone*^1^*, Marina Accardo*^2^*, Eliana Guerriero*^3^,* Federica Zito Marino*^2^*, Michele Orditura*^4^*, Alfredo Budillon***^1,§,*^***, Susan Costantini***^1,§,*^*

*^1^Experimental Pharmacology Unit, Laboratori di Mercogliano, Istituto Nazionale Tumori – IRCCS – Fondazione G. Pascale, Napoli, Italia*

***^2^****Department of Mental and Physical Health and Preventive Medicine, Università degli Studi della Campania "Luigi Vanvitelli", Pathology Unit, Napoli, Italia*

*^3^Istituto Tecnico Industriale (ITIS) “Guido Dorso”, Avellino, Italia*

***^4^****Division of Medical Oncology, Department of Precision Medicine, School of Medicine, Università degli Studi della Campania "Luigi Vanvitelli", Napoli, Italia*

*^#^These authors contributed equally to this work.*

^§^*These authors are co‐last authors.*

***Corresponding Authors:**

Dr. Alfredo Budillon, Istituto Nazionale Tumori – IRCCS - Fondazione G. Pascale, Via Mariano Semmola, 80131 Napoli, Tel.: +39 081-5903202, E-Mail: [a.budillon@istitutotumori.na.it](mailto:a.budillon@istitutotumori.na.it);

Dr. Susan Costantini, Istituto Nazionale Tumori – IRCCS - Fondazione G. Pascale, Via Ammiraglio Bianco, 83013 Mercogliano (Av), Tel.: +39 0825-1911729; Fax: +39-0825-1911705, E-Mail: [s.costantini@istitutotumori.na.it](mailto:s.costantini@istitutotumori.na.it)

**
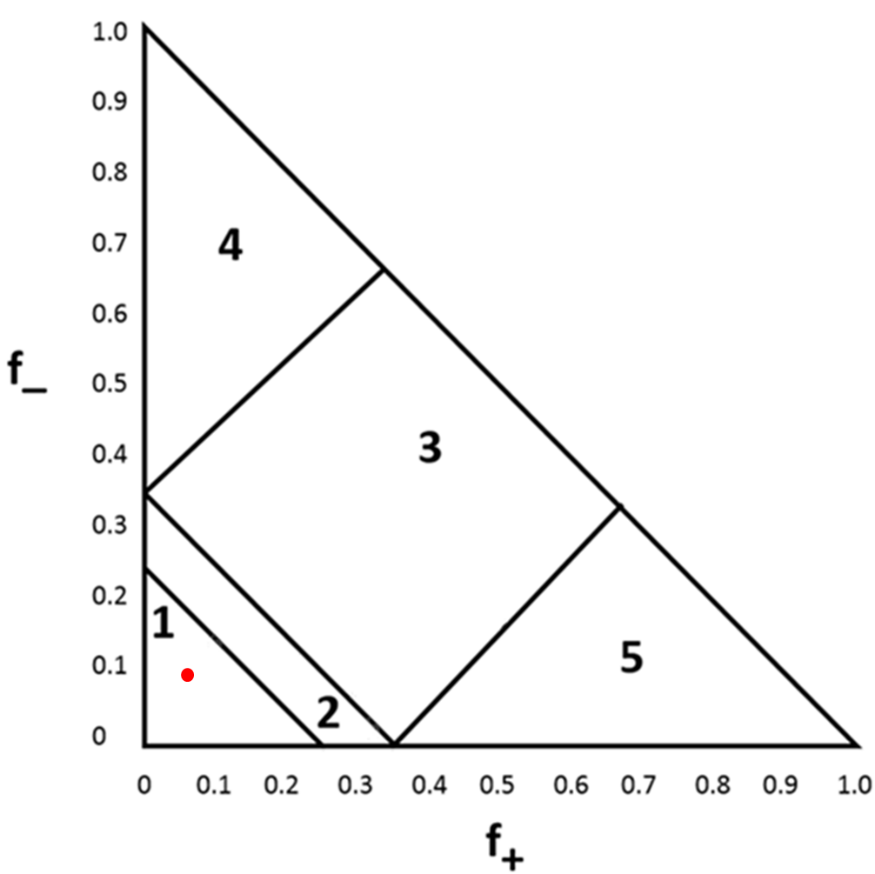
**

**Supplementary Figure 1.** State diagram obtained by Das and Pappu (2013) for SEPHS2. Thef- and f+ value for SEPHS2 is indicated by red point.

**
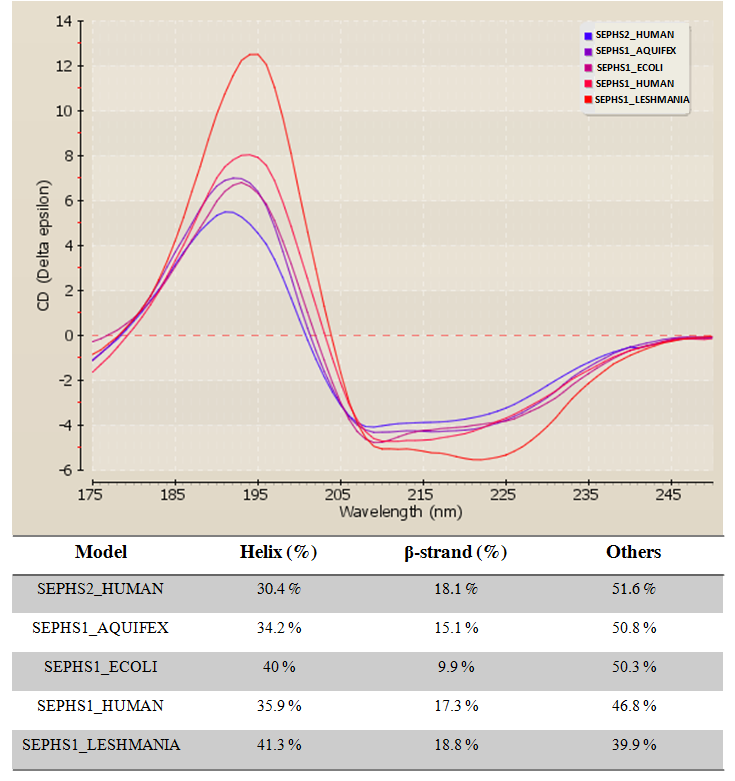
**

**Supplementary Figure 2.** Circular dichroism (CD) spectra related to our SEPHS2 model and to the crystallographic structures of *human* SEPHS1 (PDB code: 3FD5), *Escherichia Coli* SEPHS1 (PDB code: 3U0O), *Aquifexaeolicus*SEPHS1 (PDB code: 2ZOD) and *Leishmania major*SEPHS1 (PDB code: 5L16), obtained using the protein atom coordinates on PDB2CD tool (<http://pdb2cd.cryst.bbk.ac.uk/>). The percentages of secondary structure elements for each protein, evaluated by the analysis of CD spectra, are shownin the table.

A


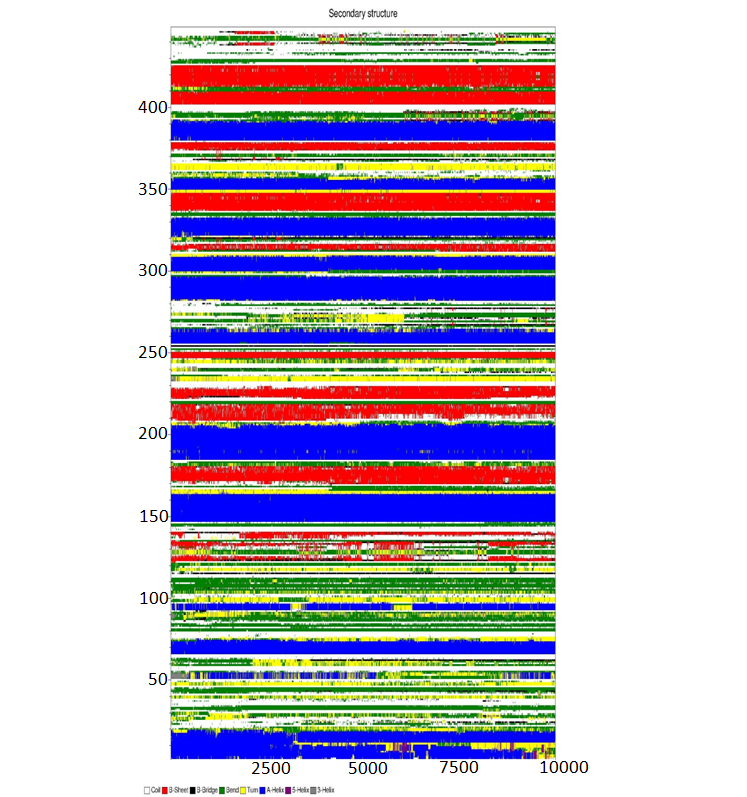


B


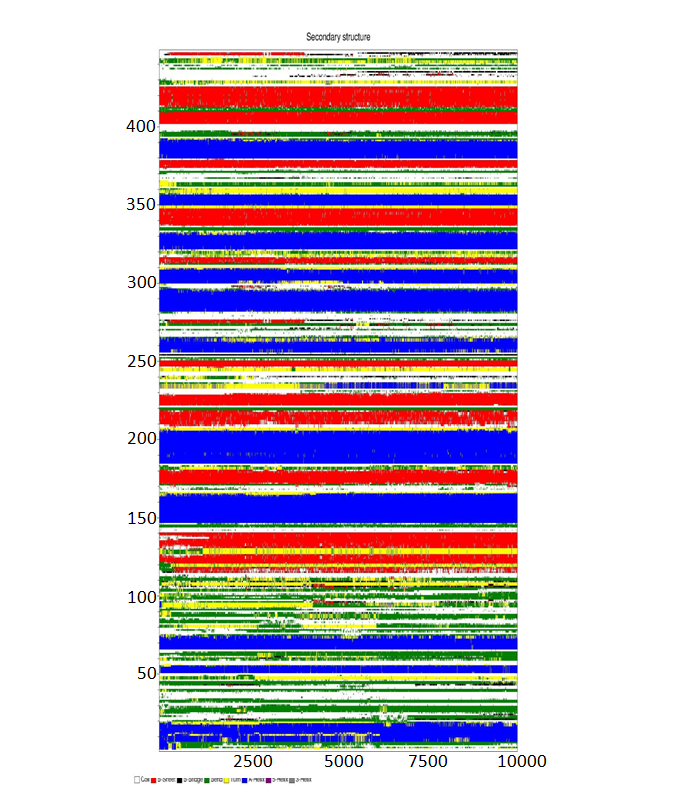


**Supplementary Figure 3.** Secondary structure evolution for SEPHS2 during MD simulation at neutral (A) acidic (B) pH. The different colorsused to evidence the type of secondary structure elements are reported in the legend.

**Supplementary Figure 4.** Covariance matrix of SEPHS2 at neutral (A) and acidic (B) pH.

**Supplementary Table 1.** Total number of H-bonds between HUB residues and the rest of SEPHS2 during MD simulation at neutral(A) and acidic(B) pH.

**A)**

| **HUB** | **0 ns** | **2 ns** | **4 ns** | **6 ns** | **8 ns** | **10 ns** |
| --- | --- | --- | --- | --- | --- | --- |
| **Phe 139** | 0 | 0 | 2 | 2 | 2 | 2 |
| **Phe 140** | 2 | 1 | 2 | 2 | 2 | 2 |
| **Leu 159** | 3 | 2 | 2 | 3 | 2 | 2 |
| **Tyr 163** | 1 | 3 | 5 | 3 | 2 | 3 |
| **Phe 199** | 2 | 2 | 2 | 2 | 2 | 2 |
| **Lys 252** | 4 | 2 | 5 | 3 | 4 | 4 |
| **Ile 322** | 2 | 1 | 2 | 2 | 2 | 2 |
| **His 325** | 2 | 5 | 5 | 7 | 4 | 4 |
| **Glu 369** | 4 | 3 | 2 | 4 | 5 | 5 |

**B)**

| **HUB** | **0 ns** | **2 ns** | **4 ns** | **6 ns** | **8 ns** | **10 ns** |
| --- | --- | --- | --- | --- | --- | --- |
| **Phe 140** | 2 | 2 | 2 | 2 | 2 | 2 |
| **Asn 157** | 0 | 0 | 0 | 0 | 0 | 0 |
| **Val 158** | 2 | 1 | 2 | 2 | 1 | 2 |
| **Lys 190** | 2 | 2 | 2 | 1 | 1 | 1 |
| **Phe 199** | 5 | 2 | 4 | 3 | 3 | 4 |
| **Glu 283** | 2 | 2 | 3 | 3 | 3 | 4 |
| **Met 295** | 1 | 2 | 2 | 2 | 1 | 3 |
| **Phe 320** | 1 | 0 | 2 | 2 | 1 | 2 |
| **His 325** | 2 | 2 | 3 | 3 | 2 | 2 |

**Supplementary Table 2.** ASA evaluation (expressed in Å^3^) for HUB Residues during MD simulation at neutral(A) and acidic(B) pH.

**A)**

| **HUB** | **0 ns** | **2 ns** | **4 ns** | **6 ns** | **8 ns** | **10 ns** |
| --- | --- | --- | --- | --- | --- | --- |
| **Phe 139** | 1.014 | 0.223 | 0.066 | 0.231 | 0.231 | 0.452 |
| **Phe 140** | 0.561 | 0.221 | 0.099 | 0.231 | 0.033 | 0.099 |
| **Leu 159** | 0.099 | 0.033 | 0 | 0.066 | 0.066 | 0.033 |
| **Tyr 163** | 0.045 | 0 | 0 | 0 | 0.094 | 0.033 |
| **Phe 199** | 0.066 | 0.066 | 0.033 | 0 | 0 | 0 |
| **Lys 252** | 0.292 | 0.090 | 0.099 | 0.104 | 0.132 | 0.135 |
| **Ile 322** | 0.170 | 0.049 | 0.012 | 0.240 | 0.132 | 0.135 |
| **His 325** | 0.236 | 0.255 | 0.156 | 0.147 | 0.180 | 0.213 |
| **Glu 369** | 0.136 | 0.183 | 0.136 | 0.169 | 0.066 | 0.099 |

**B)**

| **HUB** | **0 ns** | **2 ns** | **4 ns** | **6 ns** | **8 ns** | **10 ns** |
| --- | --- | --- | --- | --- | --- | --- |
| **Phe 140** | 0.462 | 0.198 | 0.198 | 0.231 | 0.198 | 0.231 |
| **Asn 157** | 0.012 | 0.111 | 0.069 | 0.062 | 0.098 | 0.074 |
| **Val 158** | 0.023 | 0.033 | 0 | 0 | 0 | 0.033 |
| **Lys 190** | 0.231 | 0.232 | 0.155 | 0.168 | 0.066 | 0.177 |
| **Phe 199** | 0.165 | 0.132 | 0.066 | 0.066 | 0.066 | 0.033 |
| **Glu 283** | 0.169 | 0 | 0 | 0.033 | 0.023 | 0 |
| **Met 295** | 0.066 | 0.101 | 0.023 | 0.047 | 0 | 0.089 |
| **Phe 320** | 0.551 | 0.179 | 0.287 | 0.146 | 0.155 | 0.155 |
| **His 325** | 0.174 | 0.123 | 0.111 | 0.066 | 0.012 | 0.095 |

**Supplementary Table 3. Amplification and mutation frequencies of SEPHS2**

| **Cancers** | **Amplication frequency (%)** | **Mutation frequency(%)** |
| --- | --- | --- |
| Adrenal | 1.09 | - |
| Bladder | 2.66 | 0.24 |
| Brain | - | - |
| Breast | 4.89 | 0.24 |
| Colon | 1.45 | 0.72 |
| Head&Neck | 0.72 | 1.08 |
| Kidney | - | 0.4 |
| Hepatocarcinoma | 0.27 | - |
| Lung | 0.88 | 0.14 |
| Neuro-endocrinal | - | - |
| Ovarian | 1.49 | - |
| Pancreas | - | 1.16 |
| Prostate | 0.6 | 0.6 |
| Melanoma | 0.22 | 1.56 |
| Mesothelioma | 4.35 | - |
| Stomach | - | 0.68 |
